# Response of Reeves’s Pheasants Distribution to Human Infrastructure in the Dabie Mountains over the Last 20 Years

**DOI:** 10.3390/ani11072037

**Published:** 2021-07-08

**Authors:** Shan Tian, Jiliang Xu, Jianqiang Li, Mingxiang Zhang, Yong Wang

**Affiliations:** 1 School of Ecology and Nature Conservation, Beijing Forestry University, Beijing 100083, China; doriatshan@163.com (S.T.); lijianqiang@bjfu.edu.cn (J.L.); zhangmingxiang@bjfu.edu.cn (M.Z.); 2 Department of Biological and Environmental Science, College of Agricultural, Life and Natural Sciences, Alabama A&M University, Normal, AL 35762, USA; yong.wang@aamu.edu; 3 College of International Education, Nanjing Forestry University, Nanjing 210037, China

**Keywords:** human infrastructure, Reeves’s pheasants, response

## Abstract

**Simple Summary:**

Human infrastructure development drives habitat loss and fragmentation worldwide. In China, rapid infrastructure development impacts the habitats of endangered species. This study assessed how the distribution of Reeves’s pheasant, an endangered species of the International Union for Conservation of Nature (IUCN) and a nationally protected species of China, was potentially affected by human infrastructure development in the Dabie Mountains, the species’ main distribution range, over the past 20 years. We found that human infrastructure became more extensively distributed throughout the study area and closer to locations where Reeves’s pheasants were detected. Our results suggest that the increased density of buildings and roads in the Dabie Mountains may have caused a negative impact on Reeves’s pheasants.

**Abstract:**

Human infrastructure development drives habitat loss and fragmentation worldwide. In China, over the last 20 years, rapid infrastructure development impacted the habitats of endangered species. To facilitate conservation efforts, studies of how human infrastructure affects the distribution of Reeves’s pheasant (*Syrmaticus reevesii*), an endangered species by the International Union for Conservation of Nature (IUCN) and a nationally protected species in China, are critically needed. We assessed how the distribution of Reeves’s pheasant was impacted by human infrastructure development over the past 20 years in the Dabie Mountains, the main distribution range of the species. We surveyed Reeves’s pheasants by direct sightings and indirect evidence through line transects which were randomly distributed in the Dabie Mountains from 2001 to 2002 and 2018 to 2019. We evaluated the variation of the roads and buildings in these areas in the last 20 years, and then modeled the relationship of the distribution of this pheasant with the road and building data from 2000 and 2017. Human infrastructure became more extensively distributed throughout the Dabie Mountains during the period, with all lands within 10 km of a road or a building. The distribution of Reeves’s pheasants became closer to the buildings and roads and there was a significantly positive relationship between the occurrence of Reeves’s pheasants and the distance to the nearest buildings and roads in 2018–2019. These results suggest that the increased density of buildings and roads in the Dabie Mountains may have caused negative effects on Reeves’s pheasants.

## 1. Introduction

As the human population expands, the development of infrastructure such as roads and buildings has become a key factor directly affecting biodiversity and species survival [1,2,3]. Current development trends indicate that urban areas will increase by 1.2 million km^2^ globally by 2030, while the total length of roads will exceed 5.5 million km by 2050 [2]. As a result, natural landscape patterns and vegetation composition are undergoing major changes [4]. Species survival is affected by many factors, such as traffic noise and habitat fragmentation. Studies have shown that the population density of many avian species is decreasing in busy areas and traffic noise also reduces the success of avian reproduction along roadways [5]. Habitat fragmentation caused by human infrastructure development leads to an increase in the number of patches and a decrease in patch area and patch quality [6,7]. Habitat loss and isolation limit gene flow within species and directly or indirectly affect biodiversity [8]. Many studies have shown that habitat loss and fragmentation are the main causes of biodiversity declines, when considering all factors driving biodiversity losses [6].

Recently, global and national plans for road development have been proposed that prioritize reducing environmental costs while maximizing benefits for socioeconomic development [2]. New infrastructure is most likely to arise in developing countries, including many regions that sustain exceptional levels of biodiversity and vital ecosystem services [9,10]. Sustainable infrastructure development that minimizes ecological costs requires more detailed assessments of its impacts on wildlife populations [11], but has traditionally been ignored for rapid development projects [12,13].

Wildlife responses to human infrastructure have long been recognized as an important indicator of biodiversity conservation [14]. Population-level studies may be required to analyze the long-term, large-scale consequences of human infrastructure [15]. However, there are many practical difficulties with such studies [16]. For example, for species severely affected by human infrastructure, it is difficult to determine whether to invest in management methods (regulating space, time, and behavior for the species) to mitigate the effects of anthropogenic interference [17], or to take a land management approach, which can reduce the intensity and/or frequency of disturbance [18]. Quantifying the impacts of human infrastructure on wildlife remains challenging, especially without precise data on how these effects vary with distance [19]. With the exception of raptors, little is known about how other avian forest species respond to infrastructure development, despite the high sensitivity to habitat disturbance of these species [17].

Reeves’s pheasant (*Syrmaticus reevesii*) is a long-tailed pheasant species that is endemic to central China. This species predominantly occurs in montane forests at altitudes of 200–2600 m [20] and conifer–broadleaf mixed forest is used preferentially in all seasons, as are mature fir plantations and shrubby vegetation, but during the breeding season, young fir (*Cunninghamia lanceolata*) plantations are used preferentially at the scale of the home range [21]. Reeves’s pheasants are largely herbivorous and generally forage in the forest edge and near the forest edge of the farmland. The food composition is complex [20], and the specific composition of their diet varies by season [22]. The breeding period of Reeves’s pheasants occurs from late March to early July [20]. The nesting peak is mostly from late April to early May. When the Reeves’s pheasants start to lay their eggs, the nests are shallow pits on the ground and there is no obvious nest material. During hatching, the belly feathers of the females constantly fall off into the nest and, by the end of hatching, the feathers will completely cover the bottom of the nests. The hatching period of Reeves’s pheasants is 26–27 days [20]. The main factors affecting the nest failure are human interference and predators such as the collared crow (*Corvus torquatus*), raccoon dogs (*Nyctereutes procyonoides*) and wild boar (*Sus scrofa*) [23].

As a threatened species, Reeves’s pheasant, used to be widely distributed and was relatively common in central China, but the distribution of this pheasant has become divided into eastern and western regions [24]. According to the report of the *First National Terrestrial Wildlife Resource Survey,* published in 2009, there were about 23,000 Reeves’s pheasants remaining in the wild in China. However, the estimation made by the IUCN was only 3500–15,000 in 2018 [25]. As a nationally protected species, Reeves’s pheasant is a flagship species for the mountainous region of central China and it is important for the continued protection of natural forest reserves. The distribution, abundance and survival of this species directly reflects the quality and conservation status of the forests it inhabits [26]. Human infrastructure has become a critical factor affecting habitat availability and population sizes for this species [27]. Due to its sensitivity to habitat change and low reproductive rates, the survival of Reeves’s pheasants is under threat from anthropogenic disturbance [20]. Owing to population declines, Reeves’s pheasant was listed in Appendix II of the Convention on International Trade in Endangered Species of Wild Fauna and Flora (CITES) in 2019 [24,28] and denoted a first class national protected animal [29].

By analyzing spatial data on human infrastructure and the distribution of Reeves’s pheasants in 2001–2002 and 2018–2019, this study quantified: (1) the temporal and spatial dynamics of Reeves’s pheasants in the context of human infrastructure development; (2) how roads and buildings affect the distribution of Reeves’s pheasants. We predicted that the impact of human infrastructure on Reeves’s pheasants is distance dependent and as the density of human infrastructure increased at the study area, the distance from the infrastructure to occurrence locations of Reeves’s pheasants became shorter and occurrence probability decreased over the past 20 years. We expected that the study results would provide important information on the status of Reeves’s pheasants and inform conservation measures, developing a basis for sustainable socioeconomic development that prioritizes the environment.

## 2. Materials and Methods

### 2.1. Study Area and Data Collection

**Figure 1:**
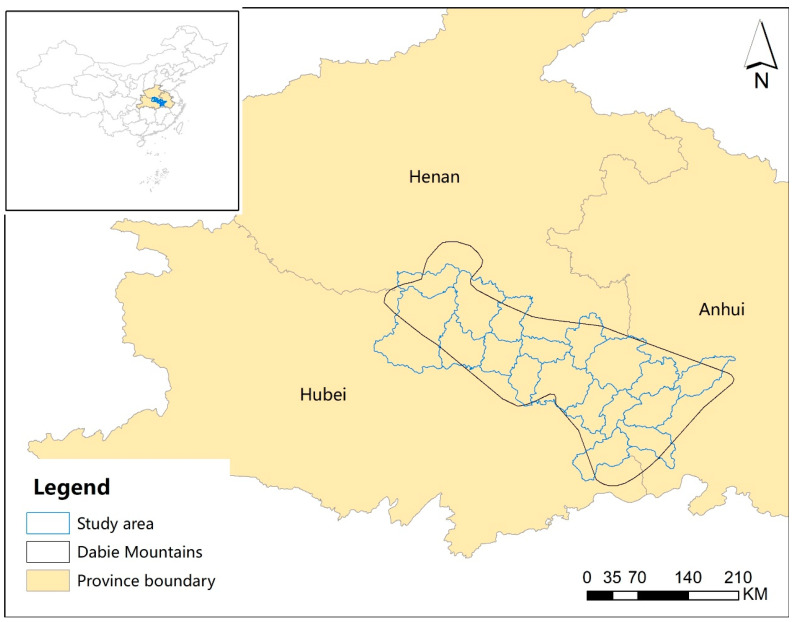
Study area in the Dabie Mountains, China. KM means kilometer.

The recent survey of Reeves’s pheasant was carried out in 2018–2019 in the Dabie Mountains, which was located along the border of the Anhui, Henan, and Hubei provinces in China, where Reeves’s pheasant populations are particularly concentrated [20,27]. A previous study of the same area in 2001–2002 enabled long-term comparisons with the present surveys [21]. To facilitate this comparison, the 2018–2019 field surveys employed the same sampling protocols established by the previous study (2001–2002), whereas the transects between the two periods were not exactly the same because of the change of the accessibility and vegetation conditions. We also revisited some additional transects where the existence of Reeves’s pheasant was reported. The study area contained seven cities and 18 districts or counties (Figure 1). Line transects varying in length from 850–3600 m were randomly distributed throughout this area and an area of 50 m to each side of each transect was surveyed for assessing the abundance by direct sightings and indirect evidence (e.g., feathers, nest sites, wing-whirring sounds, etc.) of the presence of Reeves’s pheasant. The actual length of each line transect varied due to differences in investigation time, walking speed, weather conditions, physical conditions and other factors. Surveys were carried out on foot at a walking speed of 1.2–1.5 km/h.

To avoid duplication, when a finding (feathers, feces, footprints, etc.) was recorded along a transect, other indicators observed near the location of the first finding were ignored. As the average activity distance of Reeves’s pheasants is 1.05 km [20], a radius of 1 km was used from the location of each finding to set the home range to avoid potential pseudoreplication and spatial autocorrelation. A total of 163 and 164 locations were obtained in 2001–2002 and 2018–2019, respectively.

### 2.2. Euclidean Distance Analysis for Regional Infrastructure

Human infrastructure in the Dabie Mountains is largely comprised of roads and buildings [27]. These structures are represented as lines and polygons, respectively, in a map of landscape change indicators for the region created by Sanderson et al. [9]. Road data (including railways, highways, national roads, provincial roads and country roads) of 2000 and 2017 were digitized using traffic maps for China. Building data included urban lands, rural settlements, and airports, among other factors. The building data in 2000 and 2017 were downloaded from the Resource and Environmental Science and Data Center (http://www.resdc.cn, accessed on 9 August 2018). To use these data in the current study, the data from 2000 were taken as representative of infrastructure for 2001–2002, while the 2017 data were used for 2018–2019.

In ArcGIS 10.4 (ESRI Inc. 2015, Redlands, CA, USA), the Euclidean distance tool, can be used to assess the distance between objects. A Euclidean distance map was created for all roads and buildings in the study area using this approach. By analyzing pixel data, the proportion of infrastructure pixels at different Euclidean distances from a given location can be obtained. For a given distance, the higher the proportion, the greater the amount of infrastructure in the area. For a given proportion, the smaller the distance, the more extensive the infrastructure.

The Euclidean distance to both the nearest road and building was calculated for each bird survey location for 2001–2002 and 2018–2019 separately. Using this method, infrastructure density was assessed for the Dabie Mountains for each study period and any changes in density that occurred over the past 20 years were noted.

### 2.3. Spatiotemporal Effects of Infrastructure on Reeves’s Pheasants

For each study period, the distance maps were superimposed on the distribution maps for Reeves’s pheasants. The distance between each Reeves’s pheasant locations and both the nearest road and nearest building was then calculated. The data were then summarized as the proportion of Reeves’s pheasant findings occurring at different distances from roads and buildings for both study periods.

### 2.4. Data Analysis

To understand the influence of roads and buildings on the distribution of Reeves’s pheasants, generalized linear mixed models (GLMMs) were used. We first examined multicollinearity between distances to the nearest road and building and found that multicollinearity between the predictors was not a concern (2001–2002: Tolerance = 0.806, the Variance Inflation Factor (VIF) = 1.240; and 2018–2019: Tolerance = 0.942, VIF = 1.062). The dependent variable was the presence or absence of Reeves’s pheasants, while the distances to the nearest road and building and their interactions were used as independent variables. The county (city) of each survey location was a random factor used to control for non-independence among locations. The survey findings for 2001–2002 and 2018–2019 were analyzed separately. A backward-stepwise removal method, starting with removing the nonsignificant (*p* > 0.05) interaction first, was used to simplify the initial model. When all the model terms were not significant, the significance test results of the initial model were shown (see Results).

Independent t -tests were used to assess differences in Euclidean distance to the nearest road and building for 2001–2002 and 2018–2019 in the Dabie Mountains. In addition, multivariate analysis of variance (MANOVA) was used to test the Euclidean distance between the Reeves’s pheasant locations and human infrastructure. This showed if distances to the nearest road and building differed between 2001–2002 and 2018–2019 and followed it with analysis of variance (ANOVA) to test each distance variable when MANOVA was significant. All data analyses were carried out using SPSS 21.0 (SPSS Inc., Armonk, NY, USA).

## 3. Results

### 3.1. Distance Maps for Roads and Buildings

**Figure 2:**
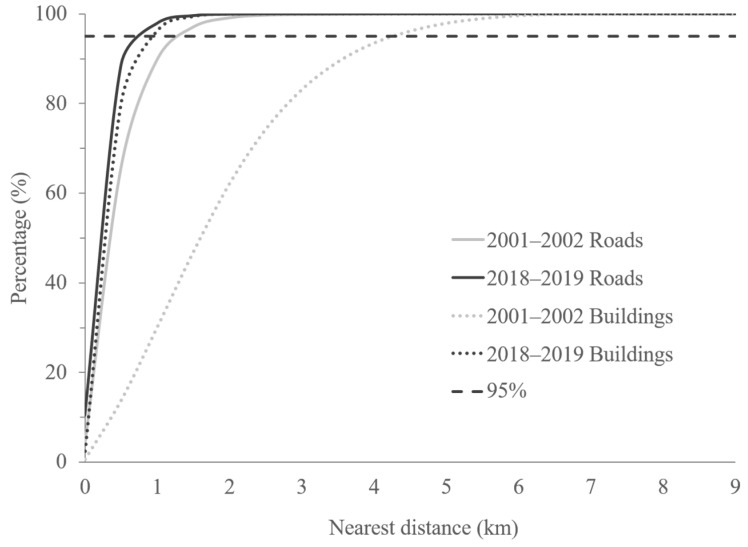
Percentage of lands by distance to the nearest road or building at the study site in the Dabie Mountains, China.

**Figure 3:**
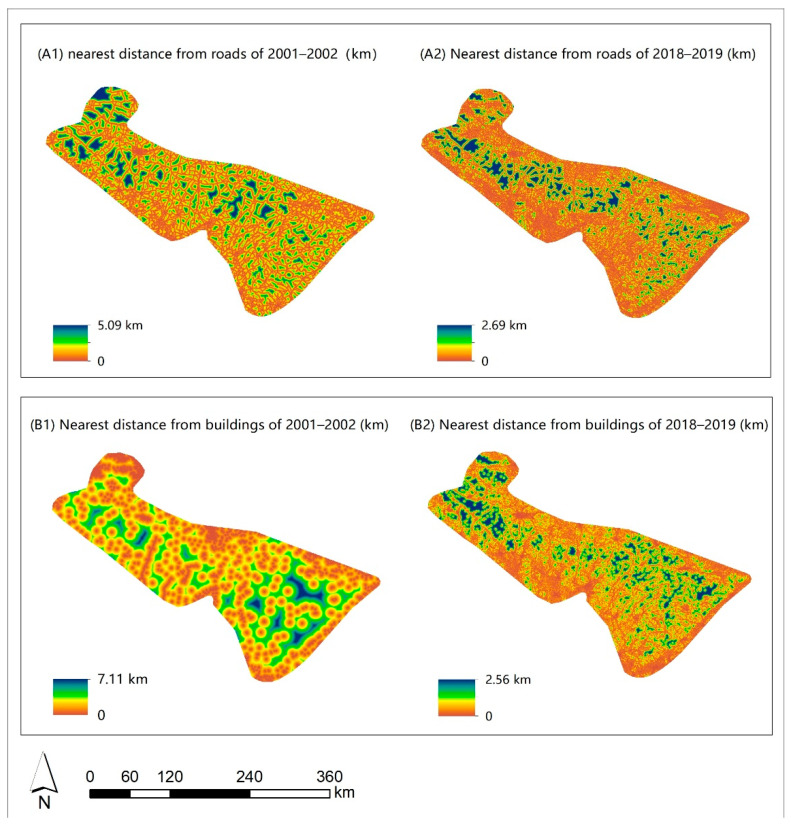
The distance to roads and buildings at the study area in the Dabie Mountains, China.

Within the Dabie Mountains, all lands were located within 10 km of a road or a building (Figure 2). The nearest road (t = 64.009, df = 6420.63, *p* < 0.001) and building (t = 140.677, df = 6620.57, *p* < 0.001) were closer on average in 2018–2019 than in 2001–2002. The maximum distance to the nearest road decreased from 5.09 km in 2001–2002 to 2.69 km in 2018–2019, while the maximum distance to the nearest building decreased from 7.11 km in 2001–2002 to 2.56 km in 2018–2019 (Figure 3). Ninety-five percent of all lands were located within 5 km of the nearest road or building (Figure 2).

Compared to 2001–2002, a higher density of roads and buildings was found in 2018–2019. In 2001–2002, ninety-five percent of all lands were located within 1.28 km of the nearest road and within 4.27 km of the nearest building; in 2018–2019, these distances decreased to 0.96 km and 0.75 km, respectively.

### 3.2. Spatiotemporal Effects of Infrastructure on Reeves’s Pheasants

**Figure 4:**
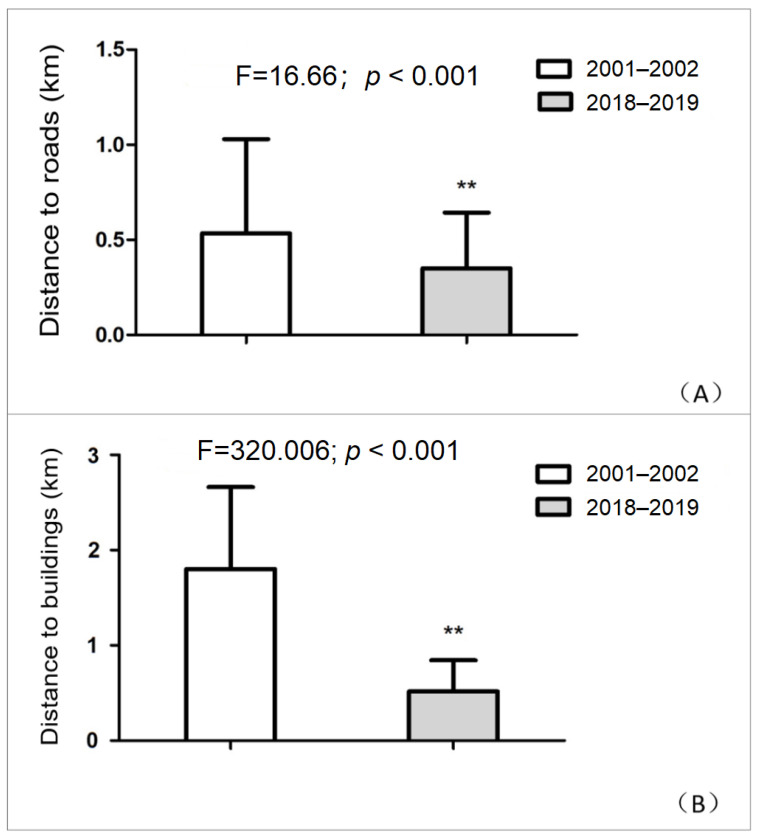
Distance between Reeves’s pheasant presence locations and the nearest (**A**) road and (**B**) building for both 2001–2002 and 2018–2019. ** *p* < 0.01.

The distance from Reeves’s pheasant occurrence locations to the nearest road and building differed between the 2001–2002 and 2018–2019 periods (MANOVA, Pillai’s trace = 0.505, F = 164.945, *p* < 0.001). The follow up test suggested that distance to the road and building became significantly shorter in 2018–2019 (F = 16.66 and 320.006, respectively, both df = 1, 325, *p* < 0.001) (Figure 4).

**Figure 5:**
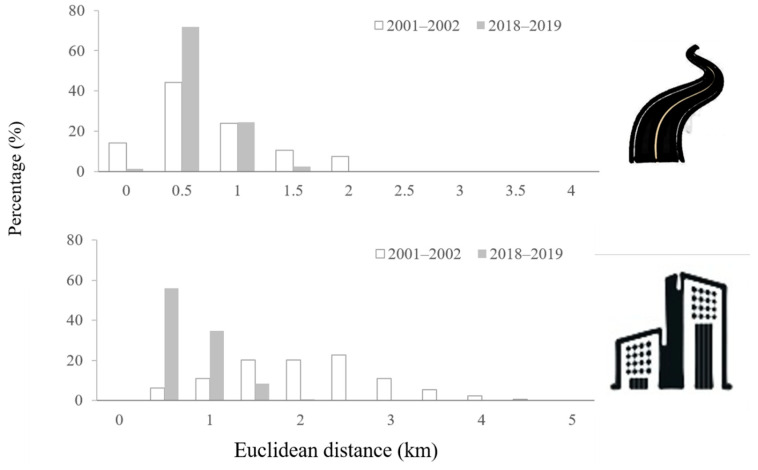
Frequency distribution of the distance to the nearest road and building of Reeves’s pheasant presence locations.

In 2001–2002, Reeves’s pheasants were most commonly located 0.5 km from a road and the same was true for 2018–2019, although the overall mean distance decreased. Reeves’s pheasants were most commonly located 1.5–2.5 km from the nearest building in 2001–2002 and 0.5–1 km from the nearest building in 2018–2019. In 2001–2002, all Reeves’s pheasants were found within 5 km of a building, while in 2018–2019, this distance decreased to 2 km. Most individuals (90.85%) were located either 0.5 km or 1 km from the nearest building in 2018–2019 (Figure 5).

The proportion of Reeves’s pheasants immediately adjacent to a road (*i.e.*, within 0 m) measured 14.11% for 2001–2002 and 1.22% for 2018–2019.

During 2001–2002, the distribution of Reeves’s pheasants was not affected by the distance to the nearest road, the nearest building, or their interaction (Table 1). In 2018–2019, the distribution of Reeves’s pheasants was significantly affected by the distance to the nearest building (Table 1). As evidenced by the positive coefficient for this factor (estimate ± SE = 3.837 ± 0.754), the nearer the closest building, the lower the probability of occurrence. The interaction was also marginally significant in 2018–2019 (Table 1). The interaction coefficient (estimate ± SE = −2.350 ± 0.128) indicated that as the distance to the nearest road increased, the effect of the distance to the nearest building decreased.

After excluding this interaction, the distance to the nearest building significantly affected the Reeves’s pheasant distribution (F_1, 325_ = 24.09, *p* < 0.001), while the distance to the nearest road was not significant (F_1, 325_ = 0.08, *p* = 0.784). This suggests that Reeves’s pheasants were more strongly affected by the presence of buildings versus roads. In addition, county had a significant effect in both 2001–2002 and 2018–2019 (Table 1), indicating that there was significant variance of Reeves’s pheasant occurrence among counties.

## 4. Discussion

The impact of human infrastructure on many wild species is distance dependent; as the density of human infrastructure increases, the distance of these structures to wildlife habitat will also decrease [2,30]. Consistent with our prediction, as human infrastructure in the Dabie Mountains grew denser, Reeves’s pheasants were found closer to infrastructure (i.e., roads and buildings). For both 2001–2002 and 2018–2019, the majority of Reeves’s pheasants occurred between 0.5 km and 1 km from the nearest road. However, in 2018–2019, the proportion of Reeves’s pheasants within 1 km of the nearest road saw an increase of over 28% compared with 2001–2002. For buildings, this effect was even more dramatic, with a higher proportion of Reeves’s pheasants occurring within 0.5–1 km of a building in 2018–2019.

Reeves’s pheasants are habitat specialists and they are highly sensitive to human disturbance [27,31]. This was evident in our results: the distance to the nearest building had a strong effect on the presence of Reeves’s pheasants. Most areas in the Dabie Mountains were located at relatively short distances from buildings and the density of buildings and roads (including at national, provincial, and county levels) increased rapidly in 2018–2019 compared with 2001–2002. Facing human infrastructure development, a species usually takes some time to respond by adapt to the new environment, move to other habitats, or even to become extinct [27,32]. Reeves’s pheasant has few options remaining, as remote areas are increasingly rare. This may also become one of the main factors causing the shortened distance between the species and the road or buildings during the two periods. As human infrastructure development increased in the Dabie Mountains, all large forested areas in the study area were subdivided by the buildings and road network [2,33] and the roads, acting as linear barriers and causing transportation disturbance, had a significant impact on the wildlife in the Dabie Mountains. This is most likely a major factor that has threatened the survival of Reeves’s pheasant [27].

Different levels of road development may have different effects on the survival of Reeves’s pheasant. The construction of national roads, provincial roads, and other urban roads has triggered a phenomenon of ‘build roads on mountains’, with roads now passing through large, forested areas. The impact of the developing road network on landscape patterns has been substantial, especially in the habitat of Reeves’s pheasants [34]. However, detailed information on the impact of forest roads on Reeves’s pheasants remains unclear and further studies are urgently needed. In this study, Reeves’s pheasants were surveyed along such forest roads for both study periods and some individuals were observed crossing the roads. This may be because these roads occurred within patches of suitable habitat of Reeves’s pheasants, with bushes, for example, distributed along the forest roads. These areas typically have high canopy density and rich food resources, thus potentially providing the protection and food for the pheasants [21]. Future research should focus on how different types of roads affect species of concern, in the context of ensuring species survival.

To effectively conserve a threatened species, it is needed to minimize the impact of human infrastructure. In the future, more action should be taken to restore habitats and establish ecological corridors to enhance the connectivity of existing nature reserves by using national parks as the main structure to protect more suitable habitats, improve the habitat integrity and reduce target species vulnerability [35,36,37].

## 5. Conclusions

In this study, the spatial and temporal dynamics of Reeves’s pheasants were examined by combining spatial and temporal data of human infrastructure with an assessment of infrastructure impacts. Human infrastructure was common throughout the Dabie Mountains, with roads and buildings becoming denser over time. In 2001–2002, 95% of lands occurred within 1.28 km of the nearest road and 4.27 km of the nearest building. In 2018–2019, these distances decreased to 0.75 km and 0.96 km, respectively. The distribution of Reeves’s pheasants became significantly related to the distance to the nearest building and road in 2018–2019: the probability of occurrence decreased as the distance decreased. Our results suggest that the increased density of buildings and roads in the Dabie Mountains may threaten the sustainability of Reeves’s pheasants.

## Figures and Tables

**Table 1:** Analysis of the effects of roads and buildings on the Reeves’s pheasant distribution using generalized linear mixed models.

Year	Variables	Coefficients ± SE	df	F	*p*
2001–2002	Distance to nearest road	1.274 ± 0.984	1, 321	1.19	0.196
	Distance to nearest building	−0.038 ± 0.281	1, 321	0.02	0.893
	Distance to road × distance to building	0.326 ± 0.445	1, 321	0.54	0.464
		Coefficients±SE		Z	*p*
	Random effect: county (city)	4.794 ± 1.823		2.63	0.009
2018–2019	Distance to nearest road	1.664 ± 0.868	1, 324	3.68	0.056
	Distance to nearest building	3.837 ± 0.754	1, 324	25.93	< 0.001
	Distance to road × Distance to building	−2.350 ± 0.128	1, 324	3.37	0.067
		Coefficients±SE		Z	*p*
	Random effect: county (city)	1.545 ± 0.689		2.24	0.025

## Data Availability

Raw data are available from the corresponding authors upon reasonable request.
